# Unraveling the Influence of Topology and Spatial Confinement on Equilibrium and Relaxation Properties of Interlocked Ring Polymers

**DOI:** 10.1021/acs.macromol.3c02203

**Published:** 2024-03-21

**Authors:** Michele Caraglio, Cristian Micheletti, Enzo Orlandini

**Affiliations:** † Institut für Theoretische Physik, Universität Innsbruck, Technikerstraße 21A, Innsbruck A-6020, Austria; ‡ Scuola Internazionale Superiore di Studi Avanzati—SISSA, Via Bonomea 265, Trieste 34136, Italy; § Department of Physics and Astronomy, University of Padova, Via Marzolo 8, Padova I-35100, Italy

## Abstract

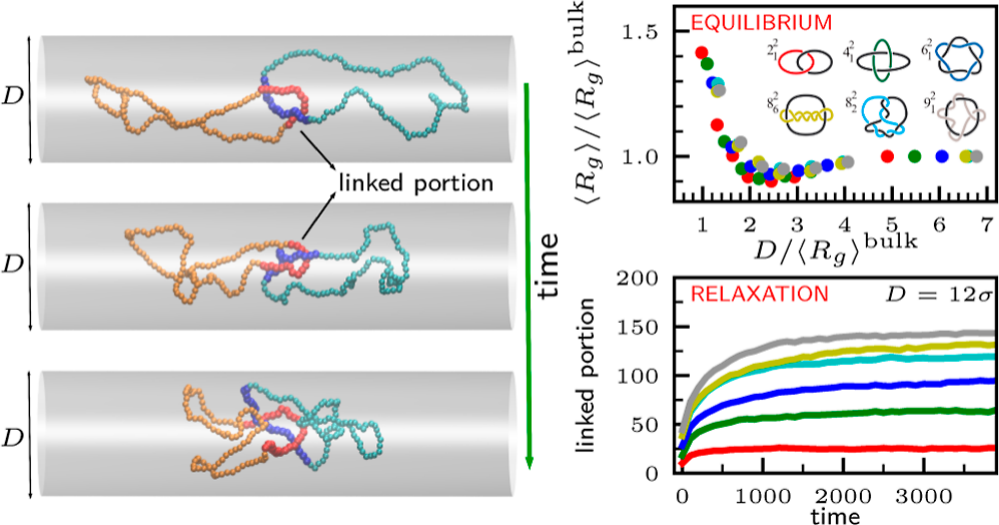

We use Langevin dynamics simulations to study linked ring polymers in channel confinement. We address the in- and out-of-equilibrium behavior of the systems for varying degrees of confinement and increasing topological and geometrical complexity of the interlocking. The main findings are three. First, metric observables of different link topologies collapse onto the same master curve when plotted against the crossing number, revealing a universal response to confinement. Second, the relaxation process from initially stretched states is faster for more complex links. We ascribe these properties to the interplay of several effects, including the dependence of topological friction on the link complexity. Finally, we show that transient forms of geometrical entanglement purposely added to the initial stressed state can leave distinctive signatures in force-spectroscopy curves. The insight provided by the findings could be leveraged in single-molecule nanochannel experiments to identify geometric entanglement within topologically linked rings.

## 1 Introduction

The emergence and time evolution of mutual entanglement between fluctuating filaments are ubiquitous phenomena in nature. They are observed in biological systems such as interlocked DNA rings in kinetoplasts,^1−4^ domain-swapped protein complexes^5^ and the intermingling of neighboring chromosomes^6,7^ and in the intricated mesh formed by polymer melts.^8−14^ Molecular linking is also receiving increasing attention because of its relevance for designing novel soft-matter systems, such as synthetic supramolecular constructs,^15−18^ Olympic gels,^18−21^ interlocked ssDNA rings,^22^ and poly[*n*]catenanes.^23−25^

Besides being interesting per se, systems of rings held together by topological constraints, also called mechanical bonds, are being studied because of their atypical properties. Striking illustrations are offered by polycatenanes of different architectures, constructs made of multiple concatenated rings.^24^ The responses of such systems to mechanical stretching, spatial confinement, translocation through narrow pores, and circularization are qualitatively different from those of conventional polymers, and so is their unique property of self-threading.^23,26−32^ Even more intriguing is their internal dynamics, both in isolation and in crowded conditions, which are significantly impacted by the concatenation constraint, e.g., via the emergence of slow modes of relaxation associated with local stiffening and length scales spanning several mechanical bonds.^24,31,33,34^

In most of the past and ongoing studies, including those outlined above, the physical implications of mechanical bonding were purposely sought in the form of collective properties, namely, through the repetition of the topological constraints. While such approaches are necessary, especially for designing extended topological metamaterials, it is equally essential to characterize how the concatenation constraint affects the behavior of the linked portion of the rings. This problem is still largely unexplored because locating and measuring the size of the physically linked regions is generally challenging. Notable exceptions are studies involving force-spectroscopy and channel confinement applied to ring pairs with the simplest linking topology, the Hopf link.^26,35,36^ Because this type of interlocking involves only two essential mutual crossings, it has been almost invariably considered the go-to interlocking motif in previous studies of systems made of mechanically bonded rings, such as poly[*n*]catenanes. Consequently, very little is known about the emerging properties conferred by other types of mechanical bonds. Based on these considerations, we consider pairs of channel-confined rings with a broad range of linking topologies. By using Langevin molecular dynamics simulations, we study how the static and dynamics of these systems depend on the link complexity and the degree of spatial confinement.

The channel-confinement setup was chosen for three main reasons: first, nanochannel confinement has become one of the leading experimental techniques to probe the statics and dynamics of (bio)polymers at the single molecule level.^37^ The setup allows for inferring various physical properties of the molecules of interest from facile measurements of their projected longitudinal span.^38−43^ The projected longitudinal overlap of multiple chains can be measured, too, making it possible to detect molecular interlockings.^44,45^ Second, channel confinement is the most convenient setting for theoretical and rigorous approaches to mutual entanglement,^46^ and it can be seen as a convenient proxy of the tubular region experienced by polymer strands within a concentrated solution of rings.^47,48^ Finally, the uniaxial elongation induced by confinement lends naturally to locating the regions where intra- and inter-chain topological entanglement is concentrated, thus allowing for tracking the evolution of physical knots and links in robust and transparent manners.^36,49−54^

Here, we use such a validated approach for pinpointing the linked region of the channel-confined interlocked rings and addressing the following questions: how does the interplay between the complexity of the topological constraint and the degree of confinement affect the system’s equilibrium metric properties? how does the relaxation time scale of pairs of linked rings depend on the complexity of the link type? how does the time dependence of the longitudinal span correlate with that of the average size of the hosted physical link? how does the relaxation dynamic of two linked rings compare to that of a single ring of equivalent contour length and to two unlinked rings that are nevertheless mutually entangled in space by mutual threading or deadlocking?

The results of our analysis are organized as follows. First, we discuss how equilibrium (static) metric properties of linked rings vary with confinement and across various families of link types, involving up to 9 essential crossings. Next, we address how the different link types relax to equilibrium from an initial stretched state again for various levels of confinement. Finally, we repeat the relaxation analysis for the more complex and realistic case where nontrivial geometrical entanglements are present on top of the topological one.

The findings help clarify the equilibrium and relaxation properties of confined interlocked rings and suggest the feasibility of using nanochannel-based experimental setups and facile measurements of the projected longitudinal span to discriminate states with low or high linking complexity.

## 2 Methods

### 2.1 Model and Simulation Setup

Pairs of topologically linked ring polymers are modeled as interlocked semiflexible circular chains, each consisting of *N* beads of diameter σ. Steric effects are accounted for by a repulsive Weeks–Chandler–Andersen (WCA) potential acting on any pair of monomers, i.e., intra- and inter-ring. As is customary, the amplitude of the purely repulsive potential was set equal to the thermal energy, ϵ = *k*_B_*T*. Consecutive beads in each ring additionally interact via a standard FENE potential,^55^ providing chain connectivity and via a bending rigidity potential, setting the persistence length to *l*_p_ = 5σ.

The two linked rings, each made of *N* = 120 beads, were confined in a cylindrical channel with periodic boundary conditions in the longitudinal direction to mimic an infinitely long channel. The excluded volume interactions of the beads with the channel walls were treated with the WCA potential.

The system was evolved with Langevin dynamics using the LAMMPS simulation package^56^ with standard values^55^ for the friction coefficient and beads mass, *m*. The integration time step was Δ*t* = 0.005τ_LJ_, where  is the characteristic Lennard–Jones simulation time.

**Figure 1:**
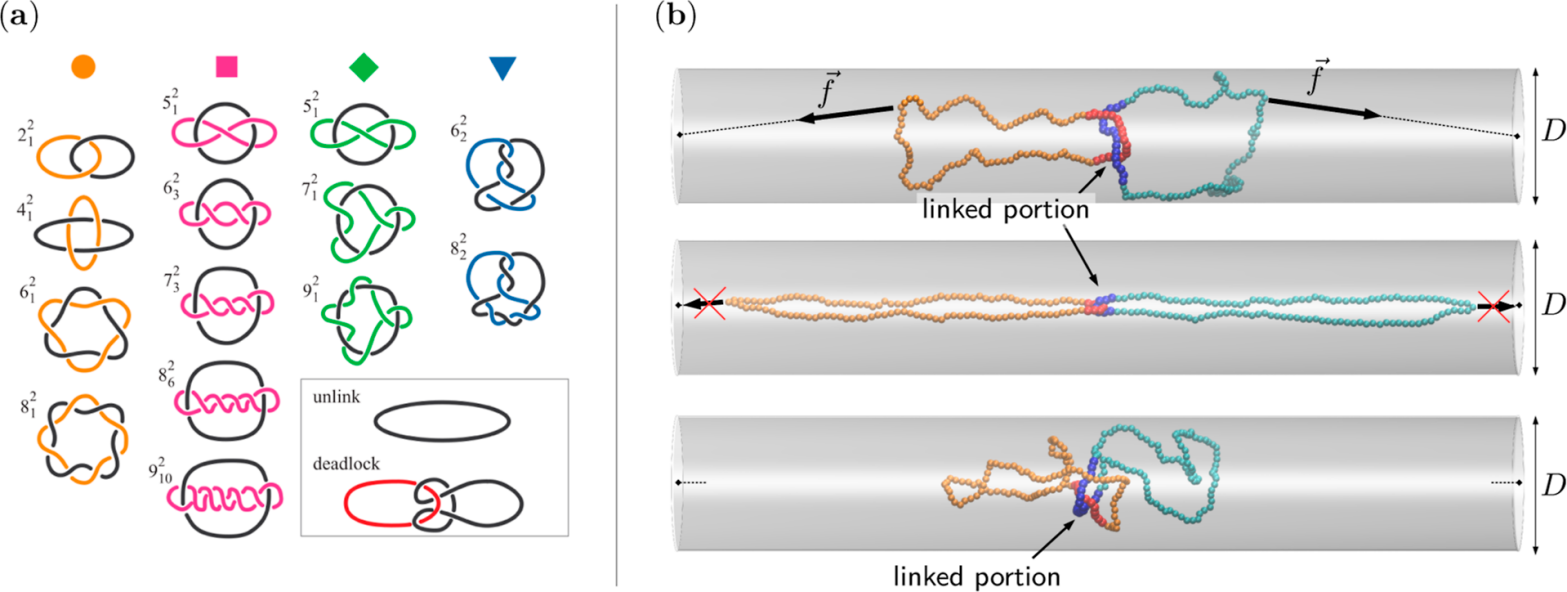
(a) Repertoire of the different topologies considered in this study. The groups correspond to torus links ({2_1_^2^, 4_1_^2^, ···}–orange–circles) and other groups having various patterns of mutual and self-crossings: twist-like links ({5_1_^2^, 6_3_^2^, ···}–magenta–squares), links with one self-crossing ({5_1_^2^, 7_1_^2^, ···}–green–diamonds), and others ({6_2_^2^, 8_2_^2^}–blue–triangles). For comparison, we additionally considered a single ring of length 2*N* and a pair of dead-locked unlinked rings. Note that the 5_1_^2^ topology appears twice because it belongs to both the second and third groups of links. (b) Preparation of the initially stretched configuration (top two panels) and its subsequent out-of-equilibrium relaxation (bottom panel).

To investigate the role of topological constraints, we considered a repertoire of 13 inequivalent linking topologies for interlocking the rings. The minimal projection diagrams of the links, which include the 2_1_^2^ (Hopf), 4_1_^2^ (Solomon), 5_1_^2^ (Whitehead), and 6_1_^2^ (star of David) links, are shown in Figure 1a, where they are labeled according to Rolfsen’s notation.^57,58^ We recall that the latter uses the crossing number, *n*_c_, i.e., the minimal number of crossings in regular projections of the curves (a projection is regular if two arcs, and no more, meet at each crossing point) as the primary descriptor; the superscript index corresponds to the number of rings forming the link (hence always equal to 2 in our systems), while the subscript is a conventional enumerative index. The topologies shown in Figure 1a cover a range of crossing numbers across four families of links, as described in the caption. Further, for comparative purposes, we considered a single (unknotted) ring of equivalent contour length 2*N* and the case of two rings, each having *N* beads, with no topological linking between them but initially entangled in a deadlocked state.

For each link type and channel width, we collected 200 independent trajectories, each of duration 2 × 10^4^ τ_LJ_, sampling configurations at 10^2^ τ_LJ_ time intervals. Each trajectory starts from an initially elongated state and reaches a fully relaxed (unstretched) state over a time span that depends on link topology and degree of channel confinement but is always shorter than 10^4^ τ_LJ_, i.e., half of the trajectory duration. We refer to the second part of the trajectory, i.e., for times between 10^4^ τ_LJ_ and 2 × 10^4^ τ_LJ_ as the equilibrium dynamics. For each link type, the initially elongated state was obtained by confining the linked rings in a narrow channel of width *D* = 11σ, pulling two beads, one per each ring, in opposite longitudinal directions with a constant force of *f* = 20*k*_B_*T*/σ, and allowing the confined system to equilibrate under mechanical tension, see also Figure 1b.

### 2.2 Observables

During the equilibrium dynamics and the out-of-equilibrium relaxation, we monitored the time evolution of various observables, such as the gyration radius
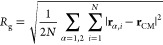
1with **r**_CM_ is the position of the center of mass and where the index α refers to one of the two rings forming the link, while the index *i* refers to the specific bead of ring α (*i* = 1, ···, *N*). We further monitored the span along the *x*-axis, which is the longitudinal direction of the channel, *s* = max_α,*i*_{*x*_α,*i*_} – min_α,*i*_{*x*_α,*i*_} and the size of the physical link, _LK_. The latter is a measure of the shortest portion of the two subchains, one for each ring, that upon a suitable closure yields the same topology as the entire link. Note that _LK_, corresponds to the smallest summed lengths of the possible combinations of linked subchains in the first and second ring. While _LK_ fluctuates limitedly in configurations at small time separations, the lengths of the two subchains may fluctuate to a larger extent and in anticorrelated manners (see Figure S1 of the Supporting Information) and for this reason are not considered. A detailed description of this observable and the algorithm used to compute it is provided in refs (26 and 59).

We calculated the equilibrium value, ⟨*A*⟩, of an observable *A* by averaging its values over 1000 conformations, considering the last five sampled for each of the 200 independent trajectories.

For the characteristic time of the equilibrium dynamics, we computed the decay time of the terminal autocorrelation function,^60,61^ τ_TACF_, which corresponds to the average reorientation time of all diameter vectors, i.e., the vectors joining two monomers at half-ring separation.^36^

The out-of-equilibrium relaxation time of an observable *A*, τ_A_, was instead obtained by integrating over time its standardized average value *f*_A_(*t*)
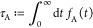
2where *f*_A_(*t*) ≔ (⟨*A*⟩_t_ – ⟨*A*⟩)/(⟨*A*⟩_0_ – ⟨*A*⟩). The notation ⟨*A*⟩_t_ ≔ ⟨*A*(*t*)⟩ refers to the average over the trajectories of observable *A* at time *t*, with *t* = 0 corresponding to the instant when the mechanical tension applied to obtain the initial configuration is switched off. Practically, the numerical integration in eq 2 is carried out from *t* = 0 to the shortest time at which *f*_A_(*t*) falls below 0.02. For all of the topologies considered, this happens earlier than *t* = 10^4^ τ_LJ_.

## 3 Results

### 3.1 Static Properties

#### 3.1.1 Links in Bulk

As a term of reference, we first considered the equilibrium properties of the linked rings with no applied force and without confinement (bulk). For the overall metric properties, we considered the equilibrium mean radius of gyration  and the average span projected on a random axis, ⟨*s*⟩^bulk^, while to characterize the entangled region, we considered the average contour length of the linked portion, .

**Figure 2:**
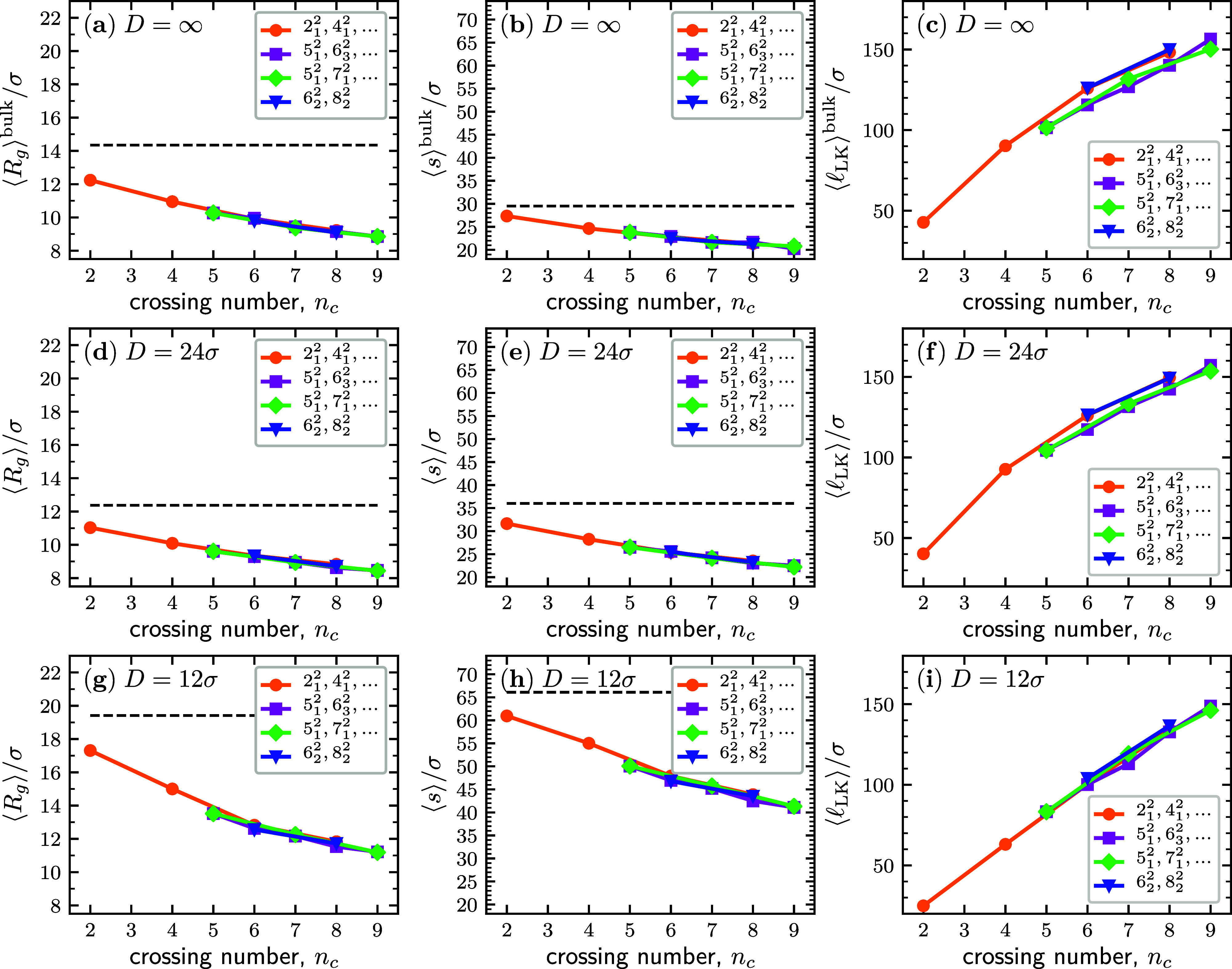
Topological dependence of the static equilibrium properties of linked rings: mean radius of gyration (a), average longitudinal span (b), and contour length of the linked portion (c) as a function of the crossing number, *n*_c_ for linked rings, each of *N* = 120 beads, at equilibrium. For comparison, the horizontal dashed lines in the panels of the first two columns refer to the case of a single ring with a contour length of 240σ. The superscript “bulk” in panels (a–c) is used to stress that these observables were computed without spatial confinement, corresponding to infinitely wide channels *D* = ∞. Panels (d–f) and panels (g–i) are equivalent to panels (a–c) but for linked rings confined in channels of diameter *D* = 24σ and *D* = 12σ, respectively. Errors bars, computed as the standard deviation of the mean, are smaller than the symbols’ size.

The metric- and topology-related observables of different link types in bulk are reported in Figure 2a–c as a function of their crossing number *n*_c_, a nominal measure of topological complexity. The data show that the longitudinal span and gyration radius decrease with the link complexity; see Figure 2a,b, while the average contour length of the linked portion increases; see Figure 2c. The behavior is consistent with previous results for stretched links.^26,59^ It can be rationalized by noting that the contour length sequestered by topological constraints (i.e., the minimal contour length required to realize a given link type) grows with topological complexity. As a result, the rings involved in complex links have a smaller effective contour length and hence a smaller gyration radius and span, too.

The remarkable feature emerging from Figure 2a is that the  data of all link types collapse onto the same universal curve. The same applies to the  data in panel b. Thus, the crossing number is a crucial order parameter for recapitulating the equilibrium properties of interlocked rings across disparate topologies, regardless of the breakdown of *n*_c_ into intra- and inter-ring contributions. For instance, the collapsed data for *n*_c_ = 6 pertain to three distinct topologies, namely, the 6_1_^2^, 6_2_^2^ and 6_3_^2^ links, for which the (*x*, *y*) combinations of self-crossings (*x*) and mutual crossings (*y*) are (0,6), (0,6), and (2,4), respectively. For the considered *n*_c_ = 7 topologies, which are the 7_1_^2^ and 7_3_^2^ links, the combinations are instead (1,6) and (3,4), respectively.

Differently, the data for the average contour length of the linked portion, , mainly fall on two distinct curves, depending on the number of mutual and self-crossings. One curve includes the data for torus links {2_1_^2^, 4_1_^2^, ···} and {6_2_^2^, 8_2_^2^} links, for which all crossings are mutual. Data for {5_1_^2^, 7_1_^2^, 9_1_^2^} links, which feature exactly one self-crossing, follow a similar trend but shifted to larger *n*_c_ values. It is interesting that the data points for twist-like links, {5_1_^2^, 6_3_^2^, 7_3_^2^}, having 4 mutual crossings independently of *n*_c_, lie practically on top of those of the previously mentioned set, despite involving different numbers of self-crossings.

Thus, the two  master curves are determined mainly by *n*_c_ and by whether the number of self-crossings is equal to or greater than zero. Compared to the former, the latter set has a larger , the increment about corresponding to a unit increment of *n*_c_.

The total contour length of links, 2*N*σ, is clearly an upper bound to the contour length of the linked portion, and the  of the most complex links realizable with the considered rings will approach the 2*N*σ = 240σ limit. Notably, for *n*_c_ ∼ 7, the linked portion length already exceeds 125σ, spanning most of the two rings’ contours. Thus, as *n*_c_ is increased to 10 and more crossings, the closing gap with the upper bound to  should act as a further constraint to the conformational freedom of the system, affecting both the static and dynamics of the linked rings.

#### 3.1.2 Links Confined in Channels

We now discuss how the equilibrium properties of linked rings vary when they are confined in channels of diameter *D*. The setting is arguably the simplest one for exploring how the topologically constrained rings respond to the interplay between their intrinsic length scales, *N*σ and , and an externally imposed one, *D*.

In Figure 2d–i, we report the same observables of Figure 2a–c but now for links under weak (*D* = 24σ) and moderate (*D* = 12σ) confinement. From a direct comparison with the bulk case (*D* = ∞), one notes that the *n*_c_-dependent trends of the metric properties (⟨*R*_g_⟩ and ⟨*s*⟩) at the different levels of channel confinement are the same as those for the bulk case (*D* = ∞), including the good collapse of the data onto a single (*D*-dependent) curve. Interestingly, the _LK_ data show an improved collapse with increasing confinement, falling approximately onto a straight line for *D* = 12σ.

**Figure 3:**
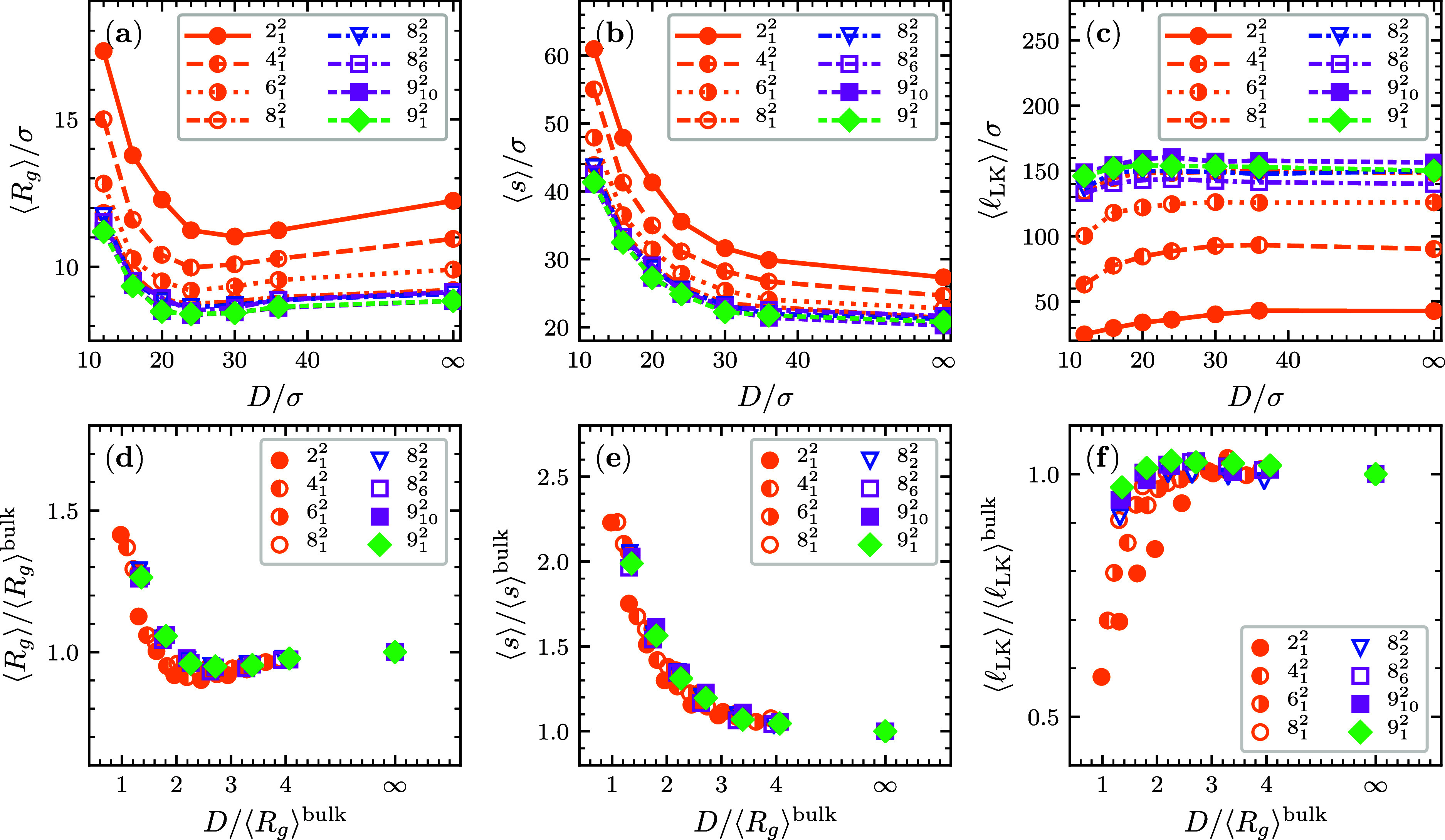
Static equilibrium properties of linked rings in channels: mean radius of gyration (a), average longitudinal span (b), and contour length of linked portion (c) as a function of channel width *D* for different link topologies. The same set of curves, but rescaled by the bulk (*D* = ∞) value are shown in panels (d–f). Errors bars, computed as the standard deviation of the mean, are smaller than the symbols’ size.

Figure 3a presents the average radius of gyration of the various link types, ⟨*R*_g_⟩, as a function of the channel width. Across all topologies, the ⟨*R*_g_(*D*)⟩ curves are nonmonotonic, with a minimum for channel widths, *D* = D*, that decreases with the link complexity.

The nonmonotonicity of ⟨*R*_g_(*D*)⟩ has been previously amply documented for channel- and slit-confined linear and ring polymers, where it emerges from the opposite monotonicities of the transverse (decreasing with *D*) and longitudinal (increasing with *D*) components of *R*_g_. For these systems, the minimum systematically occurs for D* equal to about twice  across a broad range of chain lengths and knot types.^40,62,63^

It is noteworthy that an analogous relationship holds for linked rings irrespective of their topology, as demonstrated by the data in Figure 3d where both the ⟨*R*_g_⟩ and *D* axes are rescaled by .

The approximate collapse of the data points suggests that the average span of unconstrained linked pairs is the relevant length scale to compare with the corresponding metric properties under the considered levels of confinement.

Panel (b) of Figure 3 reveals the *D* dependence of the average longitudinal span ⟨*s*⟩ of the links, a metric observable directly accessible to experiments. The data show that ⟨*s*⟩ is monotonically increasing with confinement for all link types, thus extending previous results for Hopf links;^35^ in addition, at fixed *D*, the average span decreases with the topological complexity of the link. Similarly to ⟨*R*_g_⟩, the rescaled ⟨*s*⟩ versus *D* data present an approximate collapse, see Figure 3e. The emerging master curve can help with designing or interpreting experiments because it allows for predicting metric properties valid for a broad range of topologies. For instance, the rescaled plot clarifies that for , the span is twice as large as in unconstrained limit (bulk) across the different link types.

The effect of increasing confinement on the average length of the linked portion, ⟨_LK_⟩, is illustrated in Figure 3c. As *D* decreases, the linked region becomes progressively shorter. The most significant relative variations are observed for the simplest link types, those with the smallest *n*_c_. For instance, the length ⟨_LK_⟩ for the Hopf link decreases from about 40σ in bulk to ∼25σ for *D* = 12σ. Instead, the variation is only 10% or smaller for links with *n*_c_ > 7. In the rescaled plot of the panel (f), the ⟨_LK_(*D*)⟩ data are substantially scattered for . The lack of a collapse reveals that the length of the linked portion is a more complex observable than the gyration radius and longitudinal span because it is not defined by the relative magnitude of the intrinsic size of links in bulk,  and the size of the extrinsic constraint, *D*.

### 3.2 Equilibrium Dynamics of Confined Links

To address the internal dynamics, we first studied the conformational fluctuations that occur spontaneously in links that are in equilibrium at different degrees of channel confinement.

Inspired by conventional polymer systems,^61^ we used a generalized definition of the so-called terminal autocorrelation function to compute the characteristic relaxation time scales of confined links, τ_TACF_. Specifically, τ_TACF_ was computed as the characteristic decay time of orientational correlation of diameter vectors, i.e., the distance vectors joining any two beads at sequence distance *N*/2 in the same ring.

**Figure 4:**
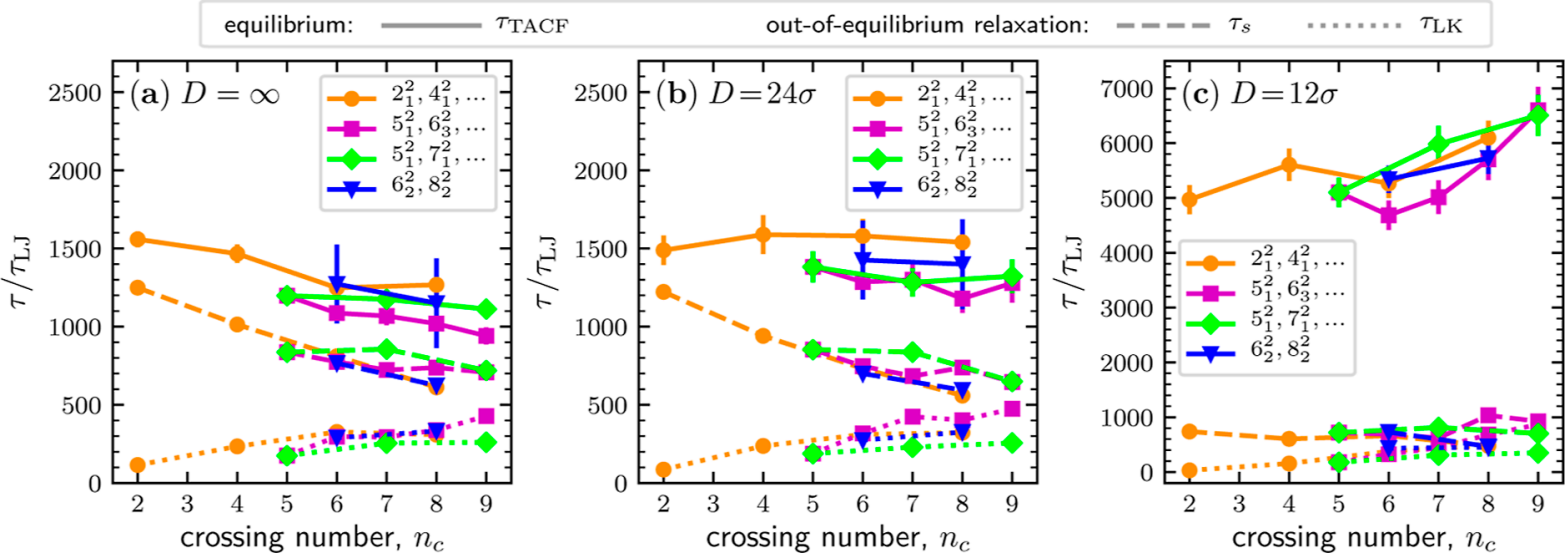
Characteristic relaxation times of confined links in- and out-of-equilibrium. For the equilibrium dynamics, we considered the orientational correlation time, τ_TACF_ (continuous line). For the out-of-equilibrium relaxation from initially stretched configurations, we considered the relaxation times of the longitudinal span, τ_s_ (dashed line), and the linked portion length, τ_LK_ (dotted line). The three panels refer to different degrees of confinement: (a) bulk, (b) *D* = 24σ, and (c) *D* = 12σ, each presenting the data as a function of links’ crossing number, *n*_c_. The error bars, which can be smaller than the symbol size, indicate the standard deviation of the mean.

The results are reported by the solid lines of Figure 4, where the τ_TACF_ versus *n*_c_ data are organized in three different panels, corresponding to as many different levels of confinement. The solid curves reported in Figure 4 establish two main results: first, the trends of the τ_TACF_(*n*_c_) curves are reversed when going from the *D* = ∞ limit (bulk) to *D* = 12σ, the tightest channels considered. In the unconstrained case, shown in panel (a), τ_TACF_ decreases with link complexity, while the opposite dependence is seen for *D* = 12σ, panel (c). Second, decreasing *D* causes a significant slowing of the internal dynamics for any given link type. For the Hopf-link, the slowing down amounts to a 3-fold increase of τ_TACF_, from ∼1550τ_LJ_ in bulk to ∼5000τ_LJ_ for *D* = 12σ. The slowing down increases with topological complexity, *n*_c_ = 9 links featuring a τ_TACF_ change from ∼900τ_LJ_ to ∼6500τ_LJ_, a 7-fold variation.

One can argue that the reversal of the τ_TACF_(*n*_c_) curves for increasing confinement results from a competition of several factors. The first one is the “drag” (topological friction), which arises because the topological constraints lead to an effective coupling of the contour sliding motion within the interlocked regions of the two rings.^36^ Counterintuitively, the topological friction grows with the length of the linked region,^36^ and hence increases with link complexity at given confinement, contributing to slowing down the dynamics with increasing *n*_c_ at fixed *D*. The second factor is the reduction of the metric size of the linked rings with link complexity (Figure 2a) due to shortening of the topologically unconstrained region. Because smaller metric footprints and contour lengths typically reflect in shorter self-diffusion and relaxation times,^48^ this size-dependent effect can speed up the relaxation dynamics with increasing *n*_c_. Finally, a third effect is the reduction of the relaxation modes accessible to the linked rings for an increasing confinement. For instance, linked rings are less and less likely to switch places along the channel as *D* diminishes. Thus, for small *D*, a tank-treading-like motion is expected to be the main relaxation mode of diameter vectors, similar to that observed in polymer rings under shear flow.^64^ This third effect thus contributes to slowing down the diameter relaxation dynamics for increasing confinement for any given topology.

With these premises, the variation of the τ_TACF_(*n*_c_) curves in Figure 4 can be ascribed to a crossover between different regimes controlled by the mentioned effects. Specifically, the decreasing trend of τ_TACF_(*n*_c_) in the bulk is consistent with more complex links having smaller sizes and thus faster Rouse-like relaxation dynamics. To corroborate this interpretation, we rescaled the τ_TACF_ data by , a quantity proportional to the nominal Rouse relaxation time. The resulting curves had a weak dependence on *n*_c_, indicative of approximate Rouse-like behavior (Figure S2). However, the approximate Rouse-like behavior of the rescaled τ_TACF_ data is lost in narrow channels (Figure S2), indicating suppression of conventional relaxation modes, with a resulting increase of τ_TACF_ with confinement, more directly illustrated in Figure S3. Note that this effect is not specific to linked rings but applies more generally and to isolated rings too (Table S1). The confinement-dependent increase of τ_TACF_ is more conspicuous for complex links, a fact that we ascribe to the increased topological friction, thus explaining the increase of τ_TACF_ with *n*_c_ in narrow channels (*D* = 12σ).

### 3.3 Out-of-Equilibrium Relaxation of Confined Links

In addition to the spontaneous internal dynamics, we addressed the out-of-equilibrium relaxation of different types of links at various degrees of channel confinement. Inspired by experimental setups where single molecules or catenated networks are stretched by mechanical forces or elongational flows,^13,65−67^ we studied the link relaxation from an initial stretched state. The latter was obtained by pulling apart the two confined rings using opposite forces parallel to the channel axis. For sufficiently large forces (see the Methods Section), the protocol yields extended states with a strongly localized linked portion at the center and a transverse footprint substantially smaller than the equilibrium value at the same channel width, *D*, see Figure 1b. The stretching forces were then switched off at *t* = 0, and the time evolution of the longitudinal span, *s*, and of the length of the linked portion _LK_ were monitored, see also Figures S4–S7 in Supporting Information. The former observable was considered because it is accessible experimentally, while the latter is informative about the dynamics of the rings’ portions directly impacted by the relaxation of the essential crossings from an initial coalesced state. For comparison, the evolution of *s* from an initially stretched single ring of equivalent size (2*N*) was considered too.

Applying eq 2 to the correlation curves for *s* and _LK_ yielded the characteristic relaxation times, τ_s_ and τ_LK_, as shown in Figure 4. The relaxation time of the length of the linked portion, τ_LK_, typically increases with *n*_c_ at all levels of confinement. Instead, the relaxation of the span, which systematically occurs on longer time scales than τ_LK_, is faster for more complex links. Indeed, at fixed *D*, the span relaxation of a single equivalent ring of length 2*N*; hence, free of topological entanglements, is typically slower than that of the linked pair of rings (see Figure S4a and Table S1). The opposite and converging trends of τ_s_ and τ_LK_ can be rationalized as follows: the increase in τ_LK_ with complexity can be ascribed to the fact that both the topological friction and the imbalance of the linked portion length of equilibrated and stretched states grow with *n*_c_. The different trends of τ_s_ and τ_LK_ can be rationalized by considering that the relative size of *s* and _LK_. For simple links, the linked region covers only a small portion of the rings so that the projected span of the system, *s*, largely exceeds _LK_. In this case, the relaxation of the linked portion will contribute only modestly to the relaxation of the system span, and hence, τ_s_ will be much larger than τ_LK_. For complex links, the linked region covers most of the rings’ contour, and the projected span of the system will be comparable to _LK_. Consequently, in this case, τ_s_ will only modestly exceed τ_LK_, explaining the convergence of τ_s_ and τ_LK_ for increasing *n*_c_. The topological underpinning of the decreasing τ_s_(*n*_c_) curves is further highlighted by plotting τ_s_ as a function of the imbalance of the linked portion length of equilibrated and stretched states, which present an approximate collapse in no and weak confinement (Figure S8).

Note also that the longitudinal span relaxation time of the simplest link types has an overall decreasing trend with confinement (Figure S3). This is reminiscent of the same decreasing trend observed for equilibrium relaxation time of the span of confined equilibrated linear Hopf-linked catenanes.^68^ However, for complex link types, the decreasing trend is reversed for *D* ≲ 20σ. Such an increase of τ_s_ with confinement appears to be a topological effect; in fact, it typically parallels an analogous increase of τ_LK_ for the more complex topologies (Figure S3).

### 3.4 Geometrical Entanglement vs Topological Entanglement

So far, we considered the relaxation dynamics of links with purely topological entanglement. By that, we mean that the mutual- and self-crossings present in the initially stretched configuration were exclusively those of the simplest, or ideal, geometrical representations of the links (Figure 1a), corresponding to the crossing number, *n*_c_.

**Figure 5:**
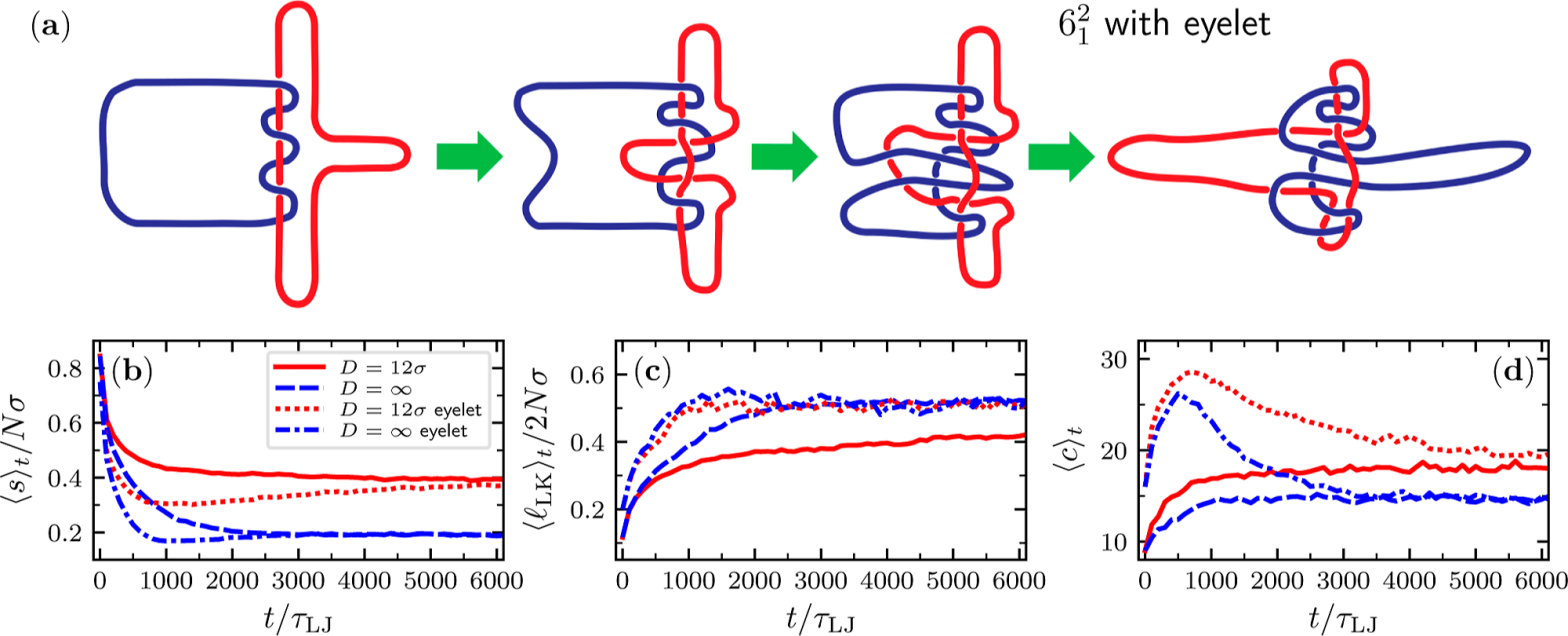
(a) Sketch of the steps followed to add geometrical entanglement (an eyelet) to a 6_1_^2^ link. The bottom panels present the time evolution of various observables for an initially stretched 6_1_^2^ link with and without an eyelet (*N* = 120) in a channel of width *D* = 12σ and in bulk. The observables are (b) average span along the channel axis and in bulk; (c) average length of the linked portion; and (d) average number of crossings. The latter average was taken over a set of 21 different planar projections with the normals to the planes picked uniformly from the unit sphere.

However, additional crossings can be introduced in the linked rings with deformations that do not involve cuts or strand passages and that, while not altering topology, can still result in more intricate geometries. An example is provided in Figure 5a, where a 6_1_^2^ link is deformed by folding the external tips of the rings inward and threading them through the mutually entangled region. This manipulation increases the mutual entanglement trapped within the link. Notice that the external tips have swapped sides at the end of the complexity-building step. Consequently, pulling the red and blue rings to the left and the right, respectively, will further entrench the added geometrical entanglement, creating a deadlocked state. Such deadlocked states have been recently reported in ring melts subject to moderate elongation flows^13^ or melts of partially active rings.^14^ Both settings create conditions conducive to the backfolding and threadings sketched in Figure 5a, which can trap even unlinked rings in long-lived entangled states.

Based on the above considerations, it is relevant and timely to consider the relaxation process of geometrically deadlocked links, where the geometric entanglement has been added and pulled taut, as sketched in Figure 5a. For reference, we will also consider deadlocked pairs of unlinked rings, see Figure 1a. Our main goal is to understand how long these geometrically entangled states last during relaxation and whether they have dynamical properties substantially different from those with purely topological entanglement. The question has clear implications for distinguishing the two types of entanglements based on the evolution of experimentally accessible observables, such as the span, and whether confinement can enhance or suppress the differences.

#### 3.4.1 Relaxation of Linked Rings Decorated with Doubly Threaded Regions

The results for the Star of David link (6_1_^2^) are presented in panels (b–d) of Figure 5. The plots illustrate the out-of-equilibrium relaxation of the stretched link prepared either in the plain linked state or with the additional doubly threaded entanglement, both with and without channel confinement; for the former, we considered the tightest channel, *D* = 12σ. The evolution is described with three different observables: the longitudinal span, the length of the linked region, and the average number of crossings, ⟨*c*⟩, namely, the number of crossings detected on a given plane projection averaged over 21 different plane projections spanning the unit sphere uniformly.

The span, *s*, shows a systematically faster relaxation to the equilibrium for the doubly threaded states (panel b), and the same holds for the length of the linked region (panel c). Interestingly, for doubly threaded states, the span has a minimum at intermediate times, an effect that we discuss further below. Instead, the ⟨*c*⟩ response (panel d) differs in two respects. First, doubly threaded states present a counterintuitive nonmonotonicity due to the ⟨*c*⟩ increase to values twice as large as the initial ones. Second, the relaxation of the doubly threaded states is now slower than the plain ones, with slowly decaying tails. Interestingly, the slowest decay is seen for the nonconfined case.

The above effects can be qualitatively rationalized with the sketches in Figure 5, which clarify that partially threaded configurations present additional crossings than in the fully developed, taut state. This is because the numerous loops of loosely threaded states project several self- and, especially, mutual crossings, unlike in the taut state. The fact that ⟨*c*⟩ presents a maximum at times comparable to those of the minimum longitudinal span is consistent with doubly threaded links being able to relax to their equilibrium configurations only after the threading loops have grown large enough to become disengaged from the entangled region.

#### 3.4.2 Relaxation of Deadlocked States of Unlinked Rings

**Figure 6:**
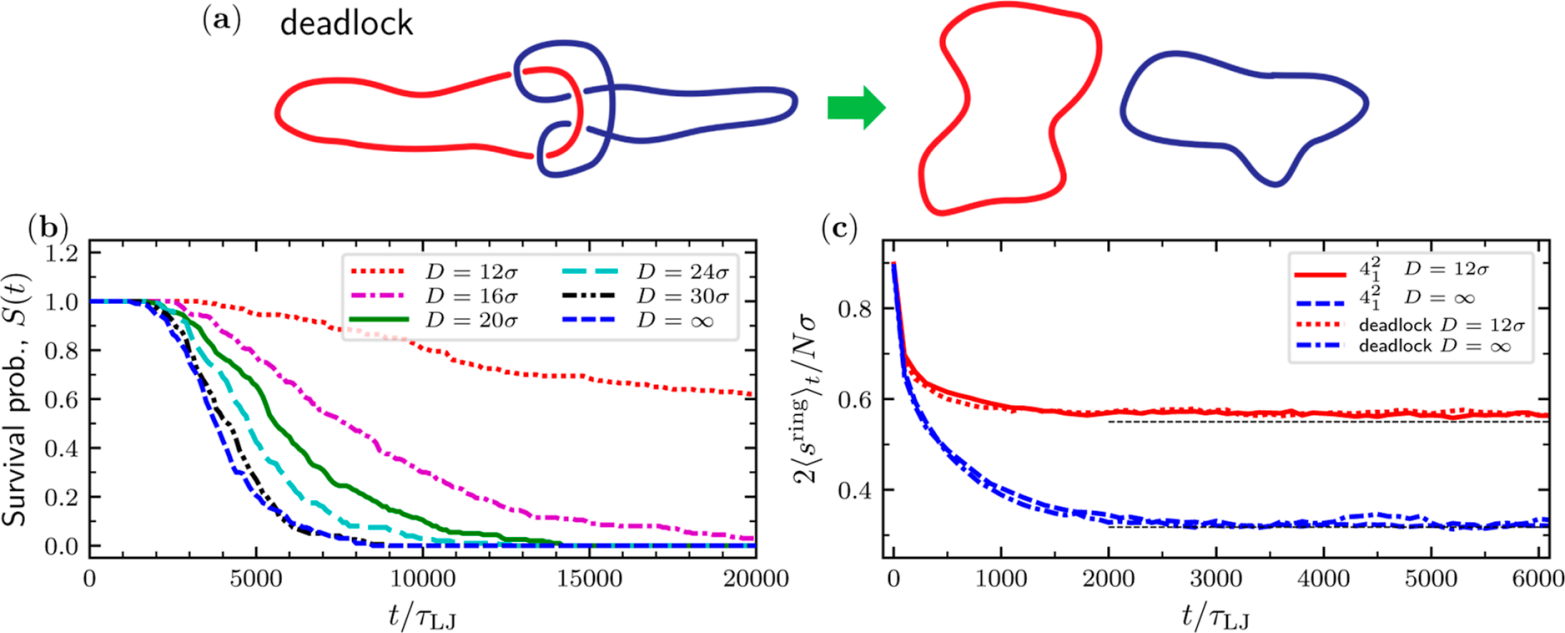
(a) Sketch of a deadlocked configuration evolving in two disjoint rings. The bottom panels present the time evolution of various observables for initially stretched deadlocked or linked rings at different confinements. (b) Survival probability of deadlocks. (c) Time evolution of the span of the single rings along the longitudinal direction in a channel of width *D* = 12σ and in bulk for the deadlock and the Solomon (4_1_^2^) link. For the deadlock, the span is computed only on trajectories still displaying a deadlocked configuration. The horizontal dashed lines indicate the equilibrium values of the observables for a single ring with a contour length 120σ.

Finally, we considered the case of two rings held together by purely geometrical entanglement, with no linking acting as a topological constraint. Specifically, we considered two unlinked rings (trivial link type, 0_1_^2^) arranged in the simplest deadlocked state, as sketched in Figures 1a and 6a. Note that although the mutual crossings generated in the deadlocked state do not result in a topological state, they are reminiscent of those in the Solomon link (4_1_^2^).

Because the rings are unlinked, they will come separated during the relaxation of the initially taut deadlocked states, an event that we detected by testing the linear separability of the links, i.e., by looking at the existence of at least one plane that fully separates in space the two rings (disengaged pairs). Note that although all linearly separable pairs of rings are disengaged, the converse is not true. In this respect, our method could overestimate the real disengagement time. However, by a visual inspection of dozens of configurations made at the sampling time just before the linear separability event (i.e., 100τ_LJ_ earlier), this case was never detected.

By gathering statistics of over 200 independent trajectories, we computed the survival probability of the deadlocked state, *S*(*t*), i.e., the probability that the rings are still deadlocked at time *t*. The results are presented in Figure 6b.

Three properties are worth noticing: (i) the initial lag time, i.e., the time at which all pairs are still deadlocked, is on the order of ∼2000τ_LJ_ that is comparable to the typical metric relaxation time of the geometrically similar link 4_1_^2^, τ_s_(4_1_^2^), as seen Figures 6c and 4; (ii) the decay is monotonic and depends sensitively on *D* becoming progressively slower with confinement. This means that channel confinement significantly hinders the simplification process of the geometrical entanglement; for instance, at the strongest confinement considered (*D* = 12σ), more than 60% of the rings are still deadlocked at times 1 order of magnitude larger than τ_s_(4_1_^2^); and (iii) at given confinement *D*, the span relaxation curves of topological linked and deadlocked states are very similar (see Figure 6c).

To quantify these observations, we fit *S*(*t*) with an exponential curve and estimate the characteristic survival times, τ_surv_(*D*) of the deadlocked states for different confinements; these are reported in Table 1. The relaxation time of the longitudinal span of the single rings, τ_s_^ring^, is also given in Table 1.

**Table 1:** Characteristic Time Scales^a^

	τ_surv_/τ_LJ_	τ_s_^ring^/τ_LJ_	τ_TACF_^deadlock^/τ_LJ_	τ_TACF_^disjoint^/τ_LJ_
*D* = ∞	4021	639 ± 6	1016 ± 66	896 ± 13
*D* = 30σ	4232	595 ± 4	1135 ± 81	842 ± 28
*D* = 24σ	5083	566 ± 4	1272 ± 45	688 ± 17
*D* = 20σ	6161	566 ± 6	1719 ± 71	1013 ± 44
*D* = 16σ	>8534	526 ± 8	3248 ± 165	1707 ± 77
*D* = 12σ	>16290	389 ± 7	4678 ± 192	4132 ± 188

a First column: diameter of the confining channel and second column: average lifetime of deadlocks, τ_surv_ ≔ , at various channel widths. Note that for *D* = 12σ and *D* = 16σ, the value of τ_surv_ is only a lower bound to the deadlock lifetime because several trajectories are unable to exit the deadlock state during the simulated time window (*t* = 2 × 10^4^τ_LJ_); third column: relaxation time of the span of the single rings in the initially deadlocked configuration, τ_s_^ring^/τ_LJ_. To calculate the equilibrium value ⟨*s*^ring^⟩ only the trajectories still in a deadlock configuration are considered; fourth column: τ_TACF_^deadlock^, decay time of the terminal autocorrelation function calculated similarly to τ_TACF_ for the usual links, but considering the time window 3 × 10^3^ ≤ *t*/τ_LJ_ ≤ 8 × 10^3^ and only the trajectories still in the deadlock state at *t*/τ_LJ_ = 8 × 10^3^; and fifth column: τ_TACF_^disjoint^, decay time of the terminal autocorrelation function calculated similarly to τ_TACF_ for the usual links, but considering the time window 1.5 × 10^4^ ≤ *t*/τ_LJ_ ≤ 2 × 10^4^ and only the trajectories already in a disjoint state at *t*/τ_LJ_ = 1.5 × 10^4^.

Comparing τ_surv_ with τ_s_^ring^, we confirm quantitatively that the purely geometrical deadlocking is highly persistent and typically lasts much longer than the relaxation process of the span, particularly in the presence of confinement. Note that this property holds even if we compare τ_surv_ with other typical time scales of the systems, such as the terminal autocorrelation function, τ_TACF_ for deadlocked and disjoint pairs of rings.

## 4 Summary and Conclusion

We used Langevin dynamics simulations to study the properties of pairs of ring polymers interlocked in different topologies and subjected to varying degrees of channel confinement. We characterized the statics and dynamics of the linked rings using conventional and experimentally accessible metric observables, such as the average span, ⟨*s*⟩, and the radius of gyration, ⟨*R*_g_⟩, as a function of channel width, *D*. In addition, we used the algorithm introduced in refs (26 and 59) to detect the ring’s physically linked portion, _LK_, and track the evolution of its contour position and length.

For rings in bulk, we found that the metric (⟨*s*^bulk^⟩ or ⟨*R*_g_^bulk^⟩) data of different link types and families all fall on the same curve determined by the crossing number, *n*_c_. More notably, *n*_c_ also indicates the longitudinal elongation of confined links. While the ⟨*s*(*D*)⟩ and ⟨*R*_g_(*D*)⟩ curves vary with topology, they all collapse on a master curve when rescaled by the corresponding bulk size.

We next studied the relaxation dynamics from out-of-equilibrium states, where the initial configurations were stretched along the direction corresponding to the channel axis. In such states, the initial length of the linked portion is significantly below the equilibrium average. While for unconstrained and weakly confined systems more complex links presented a faster decline of ⟨*s*⟩ toward the equilibrium value, this effect seems absent at the strongest confinement considered (*D* = 12σ). The setup further allowed us to explore how the longitudinal span depends on the length of the linked portion. We found a qualitatively different behavior between Hopf links, where the dependence is linear, and more complex topologies, where the dependence is compatible with a power law with effective exponents more influenced by the degree of confinement than by link complexity. This property is reminiscent of the findings of refs (69 and 70), where the relaxation dynamics of knotted DNA molecules were observed to depend more on the knot size than on the knot type.

We finally considered whether the observed relaxation properties were exclusive to proper intra- and inter-ring topological interlockings or extended to geometrical entanglements, too. Unlike topological entanglements, which are permanent, geometrical entanglements can spontaneously unravel and hence are transient, albeit possibly long-lived. We proceeded in two directions: (i) adding doubly folded threadings (eyelets) to interlocked rings and (ii) creating long-lived entangled states (deadlocks) between pairs of unlinked rings. We observed that the relaxation dynamics of the deadlocked rings were virtually indistinguishable from those of topologically linked states. At the same time, eyelets introduce nonmonotonicities in the initial part of the span versus time relaxation curve, both with and without confinement. The results indicate that the relaxation dynamics can be affected not only by topological interlockings but also by intricate geometrical entanglements and that the two contributions may be distinguishable in specific cases.

Our findings shed light on how equilibrium and dynamical properties of linked rings in channel confinement depend on the topology and geometry of their interlockings. In particular, the results suggest that facile experimental measurements of the links’ longitudinal span in nanochannels can help distinguish between different types of interlockings and thus advance the current understanding of in- and out-of-equilibrium properties of topological metamaterial and extended systems of interlocked rings. In this respect, a noteworthy system for future exploration would be kinetoplast DNA, from which clusters of linked DNA rings can be extracted and analyzed.^71^

A further interesting avenue would be exploring the role of hydrodynamic effects, which have been neglected in this first study and that might further enhance the dependence on topology of the axial diffusion coefficient, as suggested by recent experiments on knotted DNA in nanochannels.^54,72^

## Supporting Information Available

Pearson’s correlation coefficient between the linked portion located on the first ring and the linked portion located on the second ring; scaling of the orientational autocorrelation time, τ_TACF_, with respect to the radius of gyration *R*_g_; orientational autocorrelation time, τ_TACF_, and the relaxation times scales τ_s_ and τ_LK_ with respect to the confinement size *D* for different topologies; the relaxation dynamics of torus links and other topologies (Figures S4–S7); and relaxation time of the span, τ_s_, as a function of the increase of the linked portion during the relaxation process (PDF)
