# Supplementary material for: Unraveling the Influence of Topology and Spatial Confinement on Equilibrium and Relaxation Properties of Interlocked Ring Polymers
Source: Macromolecules. 2024 Mar 21;57(7):3223–33. doi: 10.1021/acs.macromol.3c02203 (PMC11008367; doi:10.1021/acs.macromol.3c02203)
Supplement: Supplementary file 1 — ma3c02203_si_001.pdf [file ma3c02203_si_001.pdf]

Supporting Information for
"Unraveling the Influence of Topology and Spatial Confinement on Equilibrium and Relaxation Properties of Interlocked Ring Polymers"

Michele Caraglio,[1] Cristian Micheletti,[2] and Enzo Orlandini[3]

[1] *Institut für Theoretische Physik, Universität Innsbruck, Technikerstraße 21A, A-6020, Innsbruck, Austria*
[2] *Scuola Internazionale Superiore di Studi Avanzati - SISSA, via Bonomea 265, 34136, Trieste, Italy*
[3] *Department of Physics and Astronomy, University of Padova, Via Marzolo 8, I-35100 Padova, Italy*

(Dated: February 27, 2024)

I. SUPPLEMENTAL FIGURES

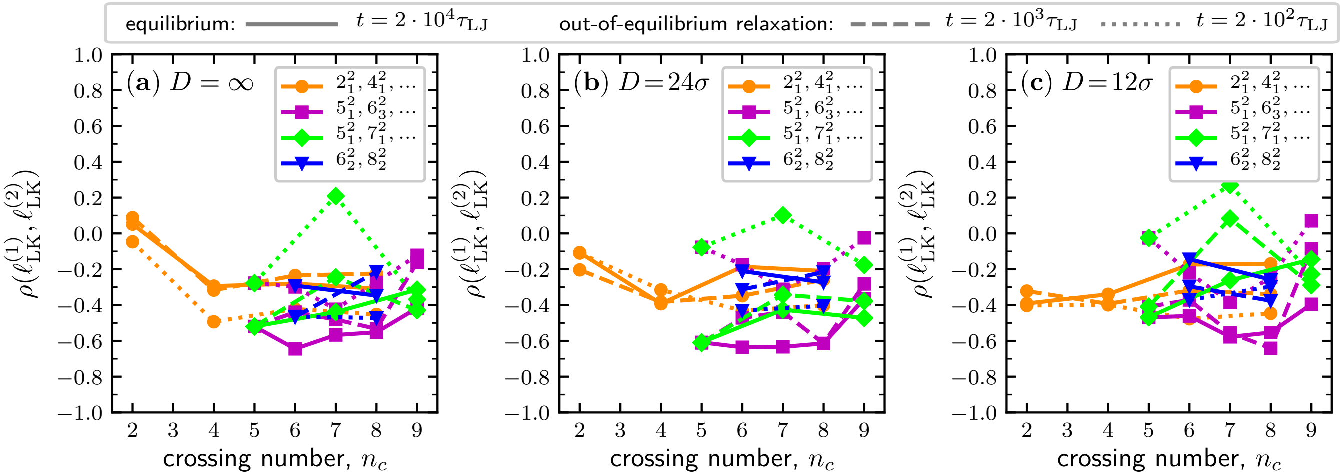

Figure S1. Pearson's correlation coefficient between the lengths of the linked portion on the first and the second ring, $\rho(\ell^{(1)}_{LK}, \ell^{(2)}_{LK})$. The correlation is calculated in two conditions: during the out-of-equilibrium relaxation (dashed and dotted lines) and at equilibrium (continuous lines). The three panels refer to the 3 degrees of confinement considered: **(a)** $D = \infty$ (bulk), **(b)** $D = 24\sigma$, and **(c)** $D = 12\sigma$. In all conditions, and for most link types, the lengths of the two linked portions are slightly anticorrelated. This is somehow expected since, in our algorithm, ℓ_{LK}, corresponds to the smallest summed lengths of the possible combinations of linked subchains in the first and second ring. While ℓ_{LK} fluctuates limitedly in configurations at small time separations, the lengths of the two subchains may fluctuate to a larger extent and in anti-correlated manners.

	τ_{TACF}/τ_{LJ}	τ_s/τ_{LJ}
$D = \infty$	3750 ± 60	1517 ± 5
$D = 30\sigma$	3582 ± 120	1518 ± 5
$D = 24\sigma$	5140 ± 180	1299 ± 5
$D = 20\sigma$	7580 ± 250	1454 ± 5
$D = 16\sigma$	12170 ± 410	1104 ± 5
$D = 12\sigma$	19010 ± 580	779 ± 7

TABLE S1. Orientational correlation time at equilibrium, τ_{TACF}, and out-of-equilibrium relaxation time of the span, τ_s for a single ring of contour length 240σ.

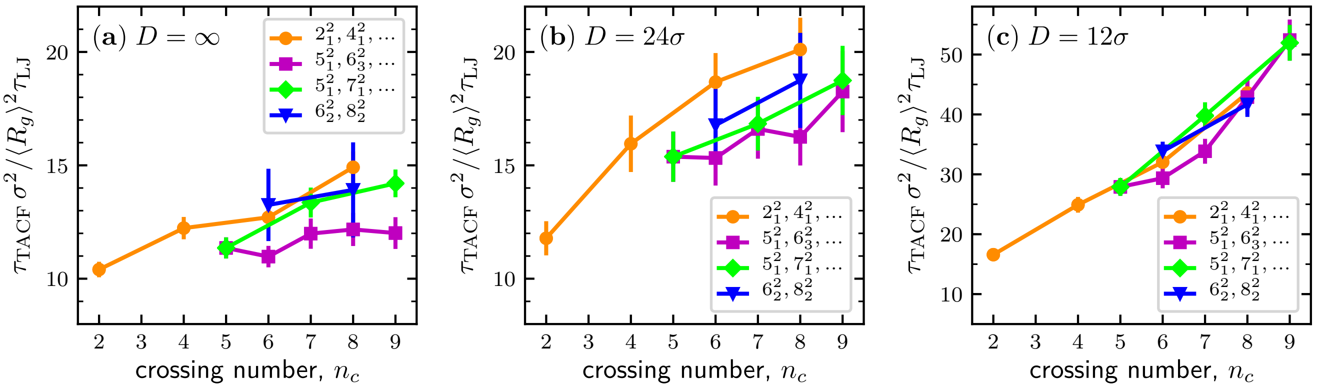

Figure S2. Orientational correlation time, τ_{TACF}, rescaled by the squared radius of gyration $\langle R_g \rangle$ as a function of the crossing number, n_c, for different link topologies. The three panels refer to the 3 degrees of confinement considered: **(a)** $D = \infty$ (bulk), **(b)** $D = 24\sigma$, and **(c)** $D = 12\sigma$. The error bars indicate the standard deviation of the mean.

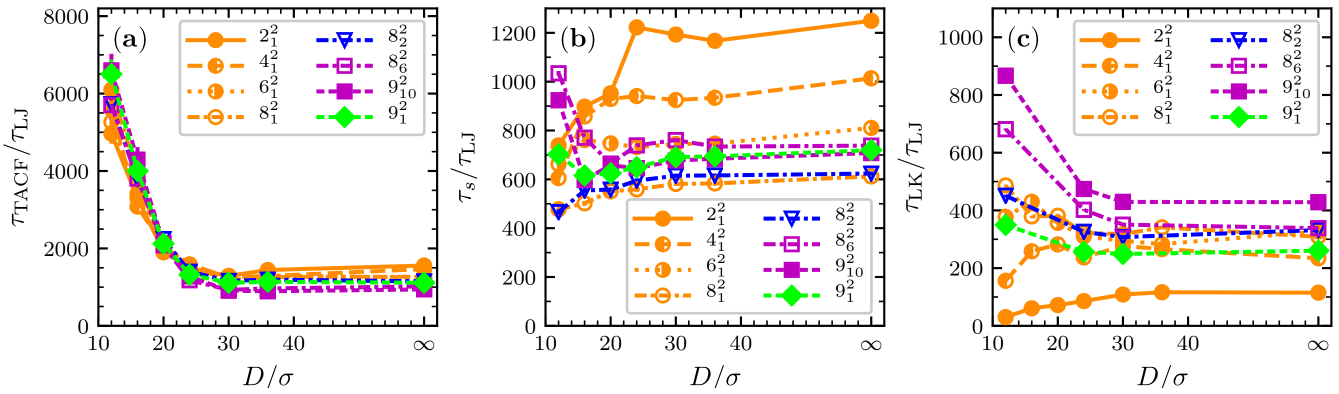

Figure S3. **(a)** Orientational correlation time at equilibrium, τ_{TACF}, **(b)** out-of-equilibrium relaxation times of the longitudinal span, τ_s, and of the **(c)** linked portion length, τ_{LK}. The data are shown for different link topologies and confinements. For a better comparison, in all panels, the bulk $D = \infty$) equilibrium values of the observables are indicated at the right-most edge of the graph.

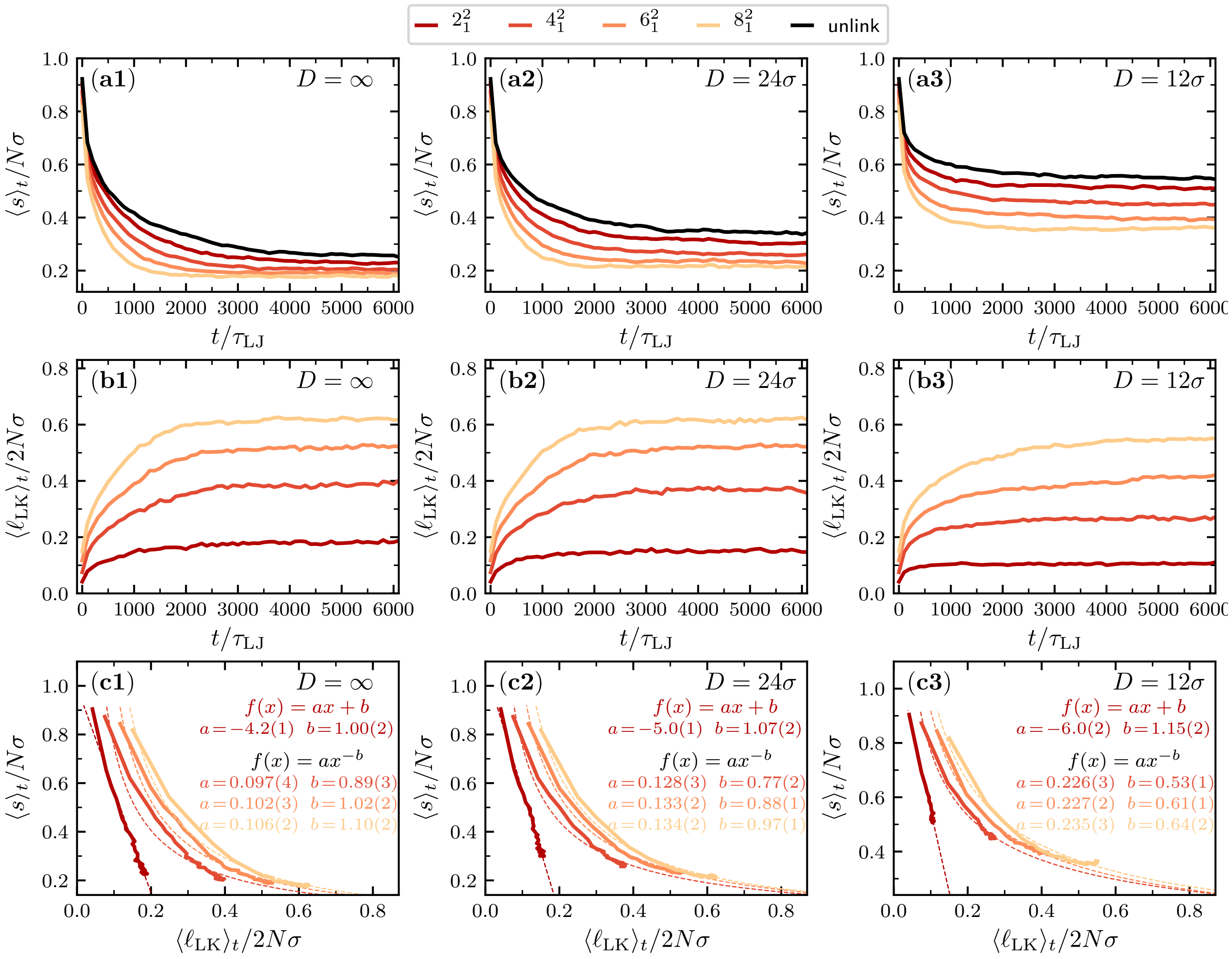

Figure S4. Out-of-equilibrium relaxation dynamics of torus links from an initially-stretched conformation in channel confinement. The relaxation dynamics is described in terms of the longitudinal span of the linked rings,**(a1-a3)** and contour length of the linked portion, **(b1-b3)**. The data are combined in the plots of panels **(c1-c3)** showing the longitudinal span's instantaneous values versus the linked portion's instantaneous contour length. Each column refers to a different value of confinement D: left Bulk, middle $D = 24\sigma$, and right $D = 12\sigma$.

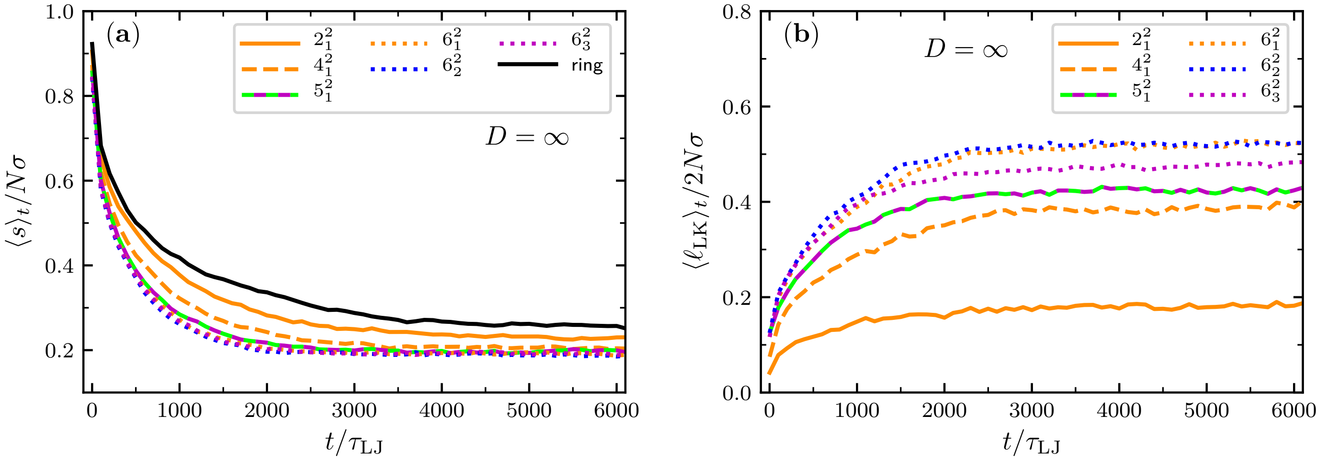

Figure S5. Out-of-equilibrium relaxation dynamics of various links from an initially-stretched conformation without channel confinement, $D = \infty$. The relaxation dynamics is described in terms of the longitudinal span of the linked rings,**(a)** and contour length of the linked portion, **(b)**. The solid black curve corresponds to a single (equivalent) ring of 240 beads.

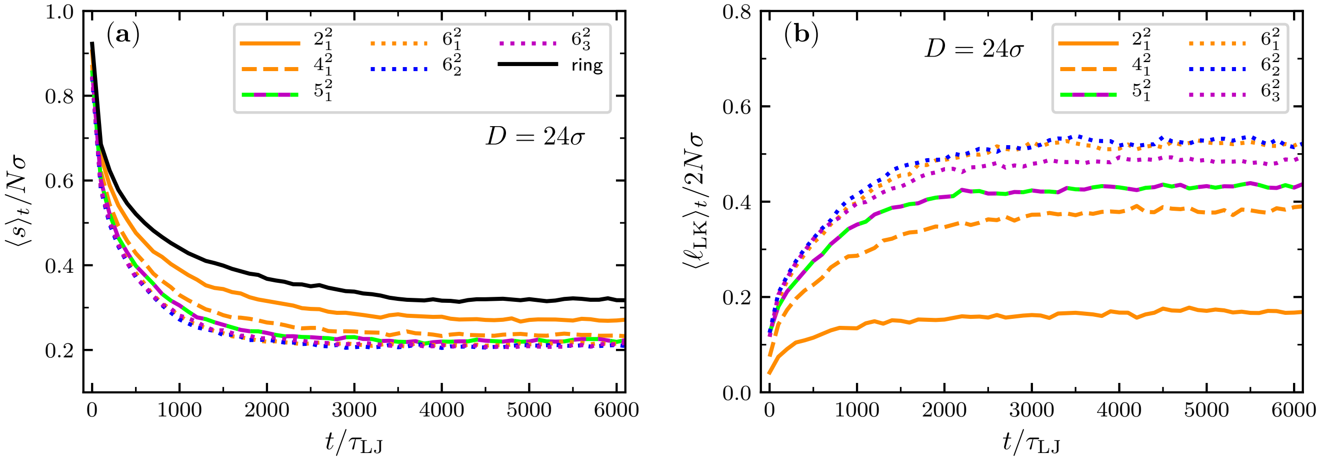

Figure S6. Out-of-equilibrium relaxation dynamics of various links from an initially-stretched conformation in channels of width, $D = 24\sigma$. The relaxation dynamics is described in terms of the longitudinal span of the linked rings,**(a)** and contour length of the linked portion, **(b)**. The solid black curve corresponds to a single (equivalent) ring of 240 beads.

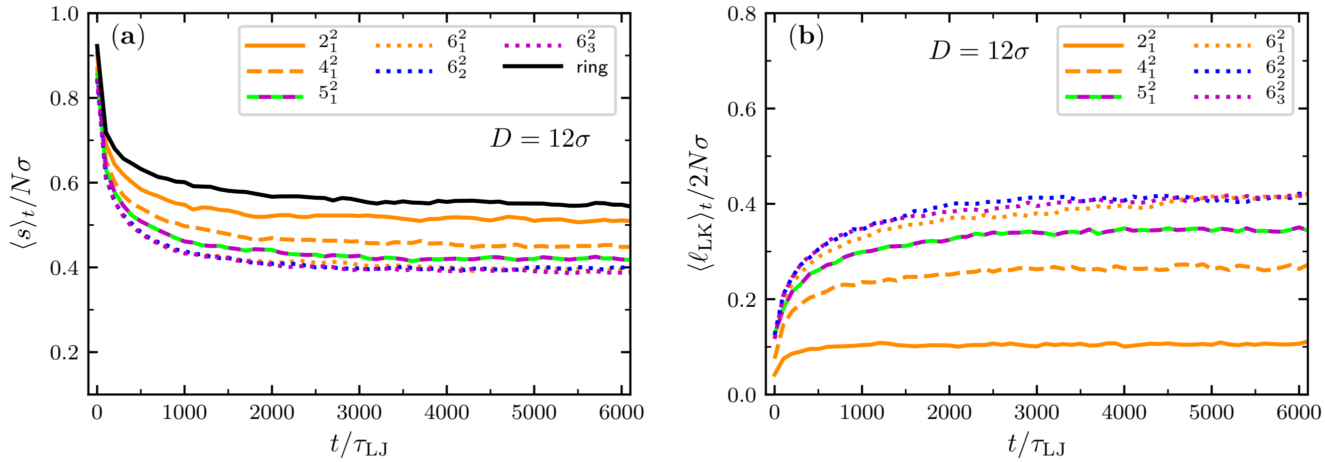

Figure S7. Out-of-equilibrium relaxation dynamics of various links from an initially-stretched conformation in channels of width, $D = 12\sigma$. The relaxation dynamics is described in terms of the longitudinal span of the linked rings,**(a)** and contour length of the linked portion, **(b)**. The solid black curve corresponds to a single (equivalent) ring of 240 beads.

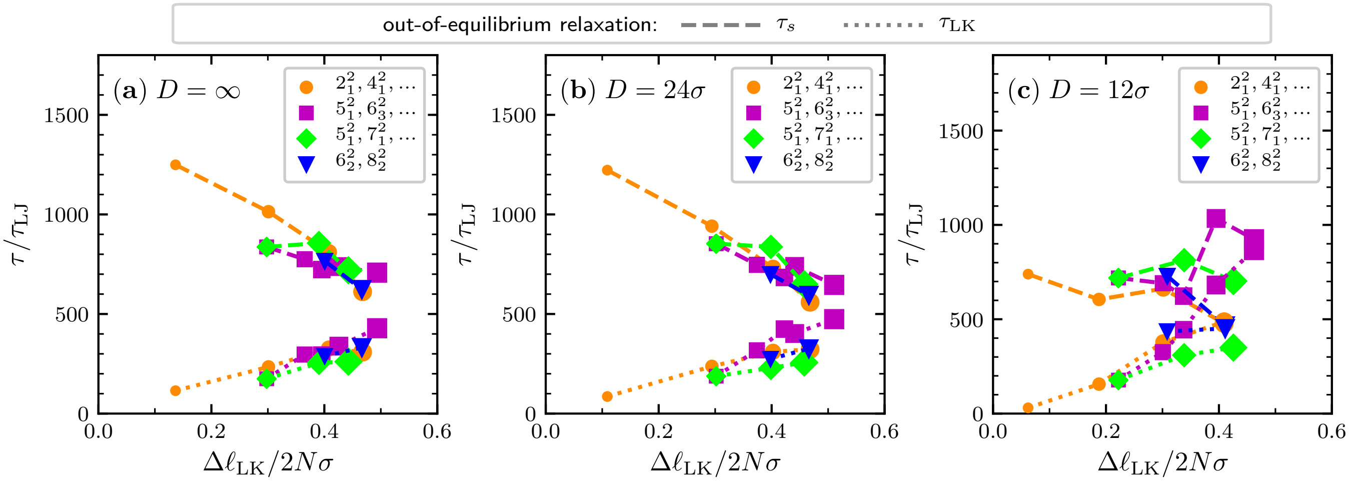

Figure S8. Out-of-equilibrium relaxation time of the longitudinal span, τ_s (dashed lines), and of the length of the linked portion, $\tau_{\rm LK}$ (dotted lines), as a function of the difference between the contour length of the linked portion at equilibrium and at the stretched initial ($t = 0$) state, $\Delta\ell_{\rm LK} = \langle \ell_{LK} \rangle - \langle \ell_{LK} \rangle_0$. The data are reported for different link topologies and confinements, **(a)** $D = \infty$ (bulk), **(b)** $D = 24\sigma$, and **(c)** $D = 12\sigma$. Members of the same topological family, are distinguished by symbol size, which increases with the crossing number, n_c.
